# A protective role of HTLV-1 gp46-specific neutralizing and antibody-dependent cellular cytotoxicity-inducing antibodies in progression to adult T-cell leukemia (ATL)

**DOI:** 10.3389/fimmu.2022.921606

**Published:** 2022-09-13

**Authors:** Yuetsu Tanaka, Reiko Tanaka, Naoki Imaizumi, Mariko Mizuguchi, Yoshiaki Takahashi, Masaki Hayashi, Takashi Miyagi, Junnosuke Uchihara, Kazuiku Ohshiro, Hiroaki Masuzaki, Takuya Fukushima

**Affiliations:** ^1^ Laboratory of Hemato-Immunology, Graduate School of Health Sciences, University of the Ryukyus, Nishihara, Japan; ^2^ Laboratory of Clinical Physiology, Graduate School of Health Sciences, University of the Ryukyus, Nishihara, Japan; ^3^ Department of Investigative Medicine, Graduate School of Medicine, University of the Ryukyus, Nishihara, Japan; ^4^ Department of Hematology, Nakagami Hospital, Okinawa, Japan; ^5^ Department of Hematology, Heart Life Hospital, Nishihara, Japan; ^6^ Department of Hematology, Naha City Hospital, Naha, Japan; ^7^ Department of Hematology, Okinawa Prefectural Nambu Medical Center and Children’s Medical Center, Minami-Haebaru, Japan; ^8^ Division of Endocrinology, Diabetes, and Metabolism, Hematology, Rheumatology (Second Department of Internal Medicine), Graduate School of Medicine, University of the Ryukyus, Nishihara, Japan

**Keywords:** ATL, HTLV-1, antibody, neutralization, ADCC, gp46

## Abstract

Human T-cell leukemia virus type-1 (HTLV-1) establishes a long-term persistent infection in humans and causes malignant T-cell leukemia, adult T-cell leukemia (ATL). HTLV-1-specific cytotoxic T lymphocytes have been suggested to play a major role in the immunosurveillance of HTLV-1-infected T cells. However, it remains unclear whether HTLV-1-specific functional antibodies are also involved in the host defense. To explore the role of antibodies in the course of HTLV-1 infection, we quantitated HTLV-1-specific neutralizing and antibody-dependent cellular cytotoxicity (ADCC)-inducing antibody levels in plasma from asymptomatic carriers (ACs) and ATL patients. The levels of neutralizing antibodies, as determined by a syncytium inhibition assay, were significantly lower in acute and chronic ATL patients than in ACs. The levels of ADCC-inducing activity were tested using an autologous pair of HTLV-1-producing cells and cultured natural killer (NK) cells, which showed that the ADCC-inducing activity of IgG at a concentration of 100 µg/ml was comparable between ACs and acute ATL patients. The anti-gp46 antibody IgG levels, determined by ELISA, correlated with those of the neutralizing and ADCC-inducing antibodies. In contrast, the proviral loads did not correlate with any of these antibody levels. NK cells and a monoclonal anti-gp46 antibody reduced the number of HTLV-1 Tax-expressing cells in cultured peripheral blood mononuclear cells from patients with aggressive ATL. These results suggest a protective role for HTLV-1 neutralizing and ADCC-inducing antibodies during the course of HTLV-1 infection.

## Introduction

HTLV-1 is prevalent worldwide, with foci of high prevalence in southwest Japan, the Caribbean islands, South America, and some areas of Central Africa and Australia (1, 2). The total number of HTLV-1 carriers is estimated to be 10–20 million (3). HTLV-1 is transmitted through contact with body fluids containing infected cells by breastfeeding and sexual contact, which occurs through cell-to-cell infection *via* the formation of virological synapses (4, 5). A characteristic of HTLV-1 infection is that most carriers remain asymptomatic throughout their lives, and approximately 5% of them develop ATL or HAM/TSP after a prolonged latency period (6, 7).

Upon latent infection establishment, HTLV-1-antigen-expressing cells are difficult to detect, at least in fresh peripheral blood mononuclear cells (PBMCs). However, when these PBMCs are isolated from the blood and cultured *in vitro*, some T cells begin to produce HTLV-1 antigen (8, 9). In addition, the continued presence of strong CD8^+^ cytotoxic T lymphocyte (CTL) responses and readily detectable levels of antibodies specific for HTLV-1 antigens (10) indicates that the persistent production of HTLV-1 continues *in vivo*. It is suggested that owing to the high degree of genomic stability (11), HTLV-1-expressing cells hardly escape from specific immune attacks by CTLs specific for Tax and HBZ antigens (12). However, the role of HTLV-1-specific neutralization and ADCC-inducing antibodies in controlling HTLV-1 infection progression *in vivo* remains unclear.

To date, several lines of evidence have shown that the major HTLV-1 envelope antigen gp46 serves as a major target for neutralization and ADCC (13, 14). The possible involvement of anti-HTLV-1 ADCC responses in the eradication of HTLV-1-expressing cells suggests that a humanized anti-gp46 monoclonal antibody (hu-LAT-27) is highly effective in the elimination of HTLV-1-infected cells in the presence of autologous natural killer (NK) cells, while preventing *de novo* infection with HTLV-1 (15). In the present study, in an effort to define the role of HTLV-1-specific antibodies with neutralizing and ADCC-inducing activities in controlling infection, we compared neutralizing and ADCC-inducing antibody levels in plasma between asymptomatic HTLV-1 carriers (ACs) and patients with ATL. Here, we show that HTLV-1 protective antibody levels were lower in patients with acute ATL than in ACs, indicating that both CTL and functional antibodies are positively involved in the immuno-surveillance of HTLV-1-expressing cells *in vivo*.

## Materials and methods

### Reagents

The medium used throughout was RPMI-1640 medium (Sigma-Aldrich. Inc. St. Louis, MO, USA) supplemented with 10% fetal calf serum, 100 U/ml penicillin, and 100 µg/ml streptomycin (hereinafter referred to as RPMI medium). The mAbs used were mouse anti-HTLV-1 Tax Lt-4 mAb (16) labeled by HiLyte Fluor 647 Labelling kit (Dojindo, Japan) according to the manufacturer’s instructions, anti-human CD25-FITC, CD16-FITC, CD56-PE, CD3-APC and anti-human CD4-PE (BioLegend, San Diego, CA), and humanized LAT-27 (hu-LAT-27) (15). HRP-labeled protein G was purchased from Abcam (Cambridge, UK). IgG was purified from the plasma using protein G (Protein G HP Spin Trap, GE Healthcare, UK).

### Blood samples

Blood samples were obtained from ACs and ATL patients living in Okinawa Prefecture after obtaining informed consent. Clinical samples were procured from seven institutions in Okinawa Prefecture (University of the Ryukyus Hospital, Heart Life Hospital, Nakagami Hospital, Naha City Hospital, Nanbu Medical Center, Chubu Tokushukai Hospital, and Kariyushi Hospital) between August 2014 and August 2021. HTLV-1 infection was confirmed by the particle agglutination method (Fujirebio, Tokyo, Japan). Patients with ATL were diagnosed based on the criteria proposed by the Japan Clinical Oncology Group and confirmed with a monoclonal integrated HTLV-I proviral genome using Southern blot hybridization, as described previously (17). PBMCs and plasma samples were separated from heparinized blood samples by density-gradient centrifugation using Lymphocyte Separation Solution (Nacalai Tesque, Kyoto, Japan) and stored in gaseous nitrogen and at −80°C, respectively, until use.

### Neutralization assay

HTLV-1 neutralizing antibody levels in samples were quantitated by performing a syncytium inhibition assay as described previously (14), with a slight modification using a new HTLV-1-producing cell line, YT-8, established from CD8^+^T cells from a normal donor by co-cultivation with the HTLV-1-producing B cell line ATL-040 cells (18). To increase the frequency of HTLV-1 gp46-expressing cells, YT-8 cells were stimulated in media containing 0.1 µg/ml prostaglandin E2 (PGE2) overnight. A volume of 5 µl of the YT-8 cell suspension at 2 × 10^7^ cells/ml in 100 U/ml IL-2 media was mixed with 90 µl of serially 2-fold diluted plasma or purified IgG in round-bottom 96-well micro-titer plates (Falcon) for 5 min, followed by the addition of an equal number of HTLV-1-negative T cell line (CEM) cells in a volume of 5 µl of the same medium. After cultivation for 4 h at 37°C in a 5% CO_2_ humidified incubator, syncytium formation was microscopically observed using an inverted microscope to determine the final dilution of plasma and IgG, which showed the complete blocking of syncytium formation. The neutralization titers of the plasma were expressed as reciprocals of the final dilutions.

### ADCC assay based on flow cytometry

The ADCC-inducing activity of antibodies was tested as described previously with a slight modification (14) using YT-8 cells and autologous NK cells. Briefly, 100 µl/well of YT-8 cell suspension (1 × 10^6^ cells/ml in 100 U/ml IL-2 medium) was mixed with the same volume of autologous NK cells (4 × 10^6^ cells/ml) selectively grown from PBMCs using an NK-cell expansion kit (KBM NK kit, Cosmo bio, Tokyo, Japan) according to the manufacturer’s protocol in duplicate in round-bottom 96-well micro-titer plates (BD) in the presence or absence of purified IgG at 100 µg/ml. After 2 days, the culture wells were examined for the number of Tax-expressing cells by FCM. Live cells harvested from the co-culture wells were Fc receptor-blocked by pre-incubation in 2 mg/ml pooled normal human IgG in FACS buffer (PBS containing 0.2% bovine serum albumin and 0.1% sodium azide) for 10 min on ice and then stained with FITC-labeled anti-CD25 mAb for 30 min on ice. After washing with FACS buffer, the cells were fixed in 1% paraformaldehyde in PBS for 10 min at room temperature, permeabilized, and washed with 0.5% saponin and 1% BSA-containing FACS buffer. The cells were then incubated with 100 µl of 0.1 µg/ml Alexa Fluor-647-labeled Lt-4 mAb for 30 min at room temperature. These cells were washed and resuspended in 300 µl FACS buffer. Cells were analyzed using FACSCalibur (BD), and the data obtained were analyzed using Cell Quest software (BD), as shown in **Supplementary Figure 2**. The absolute cell numbers in the test tubes were determined using a cell counting bead kit (Flow-Count, Beckman Coulter) according to the manufacturer’s protocol. The percent ADCC activity was calculated as follows:

(1-[CD25^+^Tax^+^ cell counts in the presence of antibody and NK cells/CD25^+^Tax^+^ cell counts in the presence of only NK cells]) × 100.

To evaluate the effect of anti-gp46 ADCC-inducing antibodies on primary T cells carrying endogenous HTLV-1, 100 µl of suspension of cryopreserved PBMCs from HTLV-1^+^ donors (1 × 10^6^ cells/ml in 100 U/ml IL-2 medium) were mixed with the same volume of medium or allogeneic NK cells (4 × 10^6^ cells/ml) in round-bottom 96-well microtiter plates (BD) in the presence or absence of hu-LAT-27 at 10 µg/ml. After 2 days, the number of CD4^+^Tax^+^ cells in the wells was examined using FCM.

### ELISA

Anti-HTLV-1 gp46 antibody levels in the plasma and IgG samples were determined by ELISA. Briefly, 50 µl of plasma diluted to 1:20 and IgG samples at a concentration of 20 µg/ml in a buffer containing 0.2% Triton X-100, 0.2% BSA, 0.05% Tween-20 in PBS was added to wells of 96-well ELISA plates (Nunc) precoated with 50 µl/well of 0.2 µg/ml affinity-purified native gp46 (14) and incubated for 1 h. After washing, the plates were incubated with 50 μl/well of HRP conjugate protein-G (Sigma-Aldrich) for 30 min. After the final wash, 50 μl/well of TMB substrate solution (Sigma-Aldrich) was added to the plates, which were incubated for 5 min, and then, the reaction was terminated by adding 50 μl/well of 2 N H_2_SO_4_. The absorbance was read at 450 nm using a reference wavelength of 620 nm with an automatic microplate reader (Bio-Rad, Hercules, CA, USA). All samples were analyzed in duplicate.

### HTLV-1 proviral loads

PVL in PBMCs were measured by quantitative PCR using 100 ng of genomic DNA (approximately equivalent to 1.0 × 10^4^ PBMCs) on an ABI 7500 Fast Real-Time PCR System (Applied Biosystems, Foster City, CA), as previously described (19). HTLV-1 PVLs were determined using the following formula: HTLV-1 tax copy number per 1.0 × 10^4^ PBMCs = ([tax copy number]/[β-actin copy number/2]) × 10^4^. All samples were analyzed in triplicate at the University of the Ryukyus Center for Research Advancement and Collaboration.

### Statistical analysis

Welch’s t-test, Kruskal-Wallis test or Spearman’s correlation analysis was performed for statistical analysis using Prism 8 software (GraphPad Software, San Diego, CA, USA). Statistical significance was set at *P* < 0.05.

### Ethical considerations

This study was approved by the Human Institutional Review Board of the University of the Ryukyus (approval number: 1470, 319-6 and 777-2). All blood samples and information were collected after obtaining written informed consent, according to the Declaration of Helsinki.

## Results

### Quantitation of HTLV-1-neutralizing antibody titers

**Figure 1:**
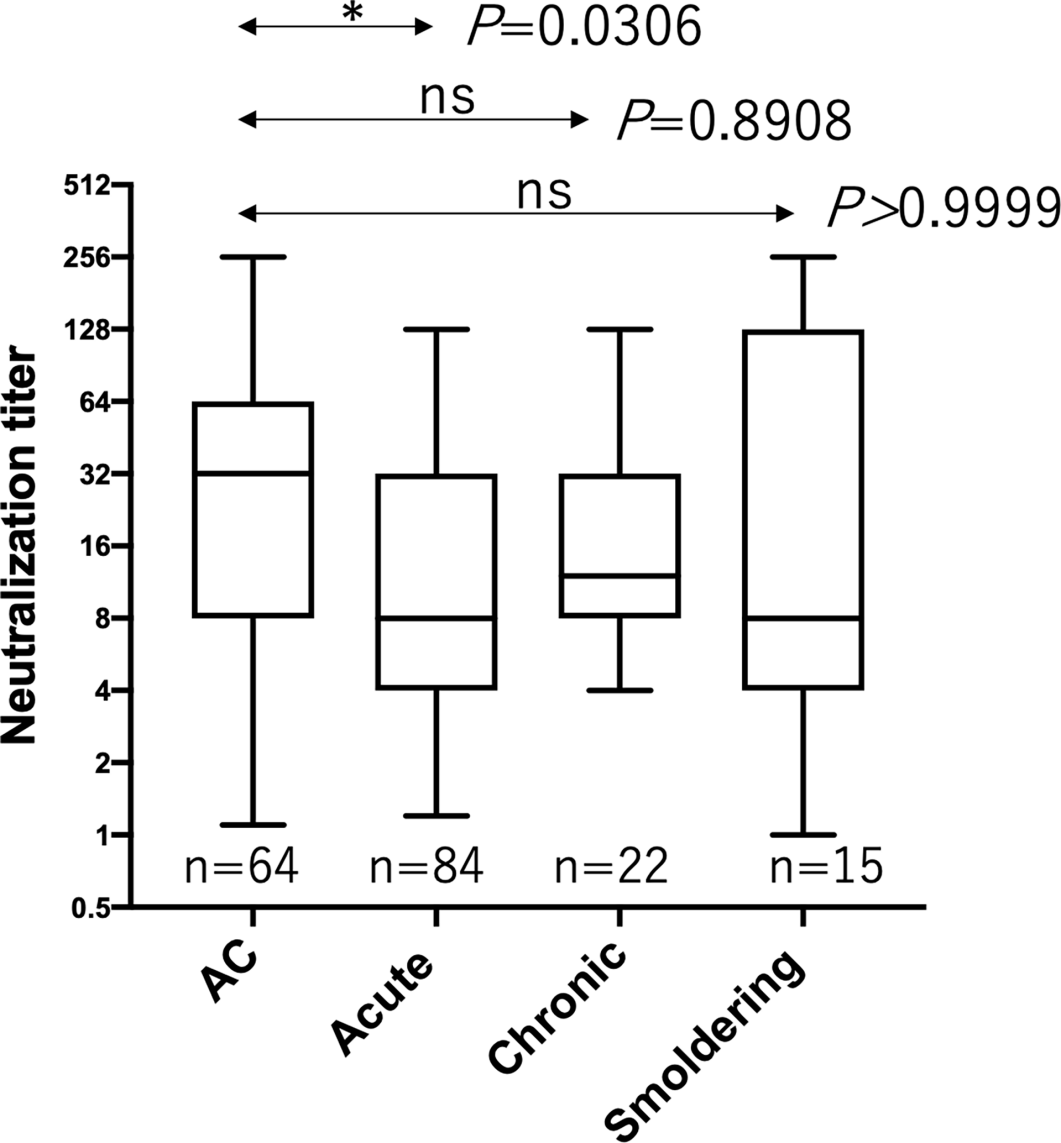
Comparison of the HTLV-1 neutralization antibody titers between ACs and ATL patients. Neutralization antibody titers were determined by a syncytium inhibition assay for ACs (n=64), acute ATL patients (n=84), chronic ATL patients (n=22), and smoldering ATL patients (n=15) and expressed as reciprocals of the final dilutions that showed complete inhibition. Statistical analysis was performed with Kruskal-Wallis test. ns, not significant.

**Figure 2:**
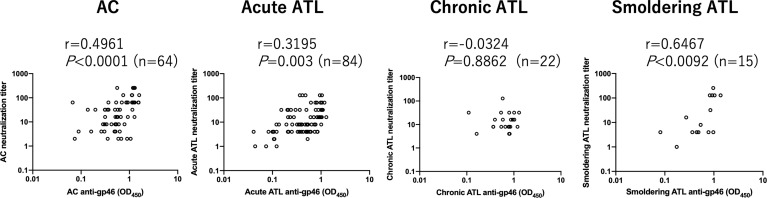
Relationship between HTLV-1 neutralization antibody titers and those of anti-gp46 based on ELISA in ACs and ATL patients. Dot plot analysis of neutralization titers (y-axis) vs. anti-gp46 titers as determined by ELISA with a 1:20 dilution of plasma (x-axis) is shown. Statistical analysis was performed with Spearman’s correlation test.

HTLV-1 neutralization levels were titrated by a syncytium inhibition assay using a pair of new HTLV-1-producing CD8^+^ T cell line (YT-8) and an HTLV-1-negative T cell line (CEM). This pair was used because syncytia induced after co-cultivation are easily recognized under an inverted microscope (**Supplementary Figure 1**). As shown in **Figure 1**, HTLV-1-specific neutralizing antibody titers of plasma from acute ATL patients, but not from chronic and smoldering ATL patients, were significantly lower than those of ACs. Simultaneously, we evaluated the levels of anti-gp46 binding antibodies by ELISA and compared them with neutralization levels. To detect total IgG antibody levels specific for the HTLV-1 gp46 antigen, a native gp46 protein, which was purified from culture supernatants of HTLV-1-producing cells (MT-2) by antibody-affinity column chromatography, was used as the immobilized target antigen. At a dilution of 1:20, the levels of anti-gp46 IgG in the plasma were clearly correlated with those of the neutralization titers in ACs, acute and smoldering ATL patients (**Figure 2**). These results showed that the levels of HTLV-1 neutralization titer and anti-gp46 antibody titer were lower in acute ATL patients than in ACs, and suggested that these neutralization activities were mainly mediated by anti-gp46 IgG antibodies, as reported previously (14).

### Quantitation of HTLV-1 ADCC-inducing antibody levels

**Figure 3:**
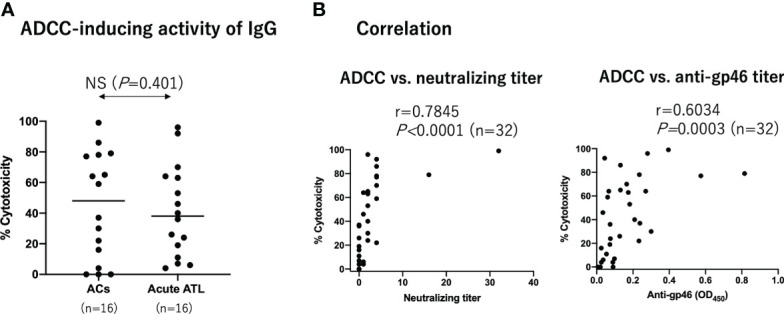
ADCC-inducing activity of IgG from ACs and acute ATL patients. Purified IgG from ACs (n=16) and acute ATL patients (n=16) at a final concentration of 100 µg/ml were added to co-culture HTLV-1 producing YT-8 cells and autologous NK cells. After 2 days, the number of CD25^+^Tax^+^ cells was determined by FCM as shown in **Supplementary Figure S3**. **(A)** Comparison of ADCC-inducing IgG activity between ACs and acute ATL patients. Statistical analysis was performed using t test with Welch’s correction. **(B)** Correlation between ADCC-inducing IgG activity at 100 µg/ml and neuralization IgG titers at 2 mg/ml, and anti-gp46 IgG ELISA titers at 20 µg/ml, among all ACs and ATL patients tested (n=32). Statistical analysis was performed with Spearman’s correlation test.

Next, we determined ADCC-inducing antibody levels using an autologous pair of target and effector cells. The target YT-8 cell line was selected because it is less sensitive to autologous NK cells than other CD4^+^ HTLV-1-producing T cell lines (Tanaka et al. unpublished). To reduce possible cytotoxic components in the plasma, we purified IgG from the plasma and tested them at a final concentration of 100 µg/ml. Samples were randomly selected from ACs (n=16) and acute ATL patients (n=16). The effector NK cells used were selectively expanded from PBMCs *in vitro*, and most of them expressed CD16 on the cell surface (**Supplementary Figure 2**). As shown in **Supplementary Figure 3**, the CD25^+^Tax^+^ cell number in each well after co-cultivation was counted by FCM, and the ADCC-inducing capacity of IgG was calculated as cytotoxicity. In these experimental conditions, NK cells at E/T ratio of 4 and 10 μg/ml humanized anti-gp46 mAb (hu-LAT-27) could achieve complete eradication of the target YT-8 cells during cultivation for 7 days *in vitro* (**Supplementary Figure 4**). **Figure 3A** provides a summary indicating that the ADCC-inducing capacity of IgG was comparable between ACs and patients with acute ATL patients. Notably, the ADCC-inducing capacity of IgG was significantly correlated with both neutralizing and anti-gp46 antibody levels among the total samples tested (**Figure 3B**). These results showed that there was no difference in HTLV-1-specific ADCC-inducing antibody levels at a fixed concentration of IgG between ACs and acute ATL patients. However, since the neutralization titers were lower in acute ATL patients than in ACs, it can be speculated that the total plasma levels of ADCC-inducing antibodies in acute ATL patients were also lower than those in ACs.

### No correlation between PVL and anti-gp46 antibody levels

**Figure 4:**
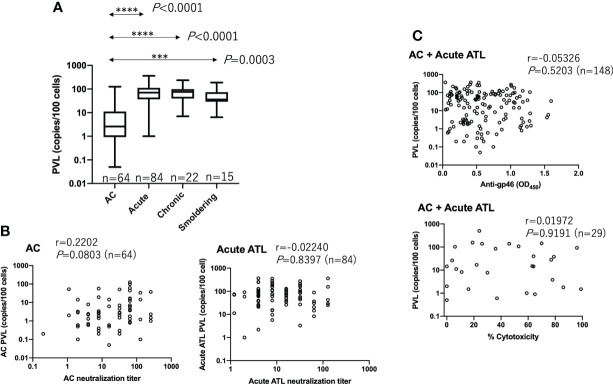
No correlation between PVL and neutralizing, ADCC-inducing, or anti-gp46 antibody titers. **(A)** PVL in each group of ACs (n=64), acute ATL (n=84), chronic ATL (n=22), and smoldering ATL (n=15) patients. Statistical analysis was performed with Kruskal-Wallis test. **(B)** Relationship between PVL and neutralizing titers in ACs and acute ATL patients, and **(C)** Relationship between PVL and anti-gp46 antibody or ADCC-inducing levels. Statistical analysis was performed with Spearman’s correlation test in **(B, C)**.

It has been reported that antibody titers against a mixture of multiple HTLV-1 antigens are correlated with the PVL (20, 21). Thus, we tested whether neutralizing, ADCC, and anti-gp46 ELISA antibody levels were correlated with PVL. The prominence of PVL was clear in the ATL samples compared to that in ACs (**Figure 4A**). However, as shown in **Figures 4B, C**, we found no significant correlation between PVL and any of the three antibody levels. These results indicated that antibody production against the gp46 antigen was independent of PVL.

### ADCC reduces the number of Tax^+^ cells in primary PBMC cultures of ATL patients

**Figure 5:**
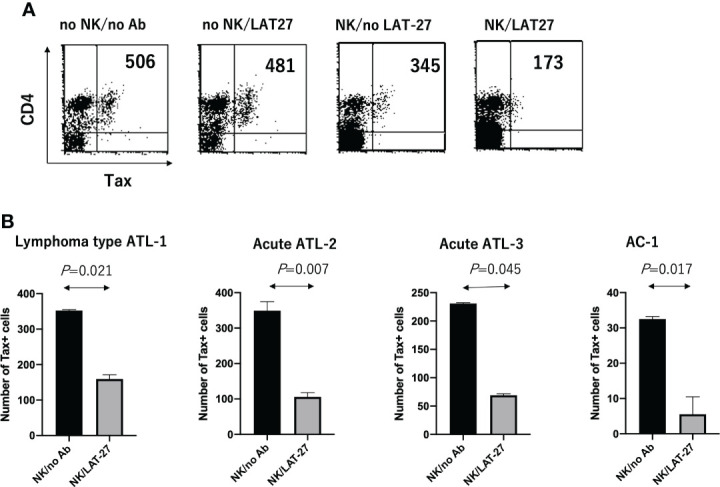
Reduction of the number of CD4^+^Tax^+^ cells by ADCC in cultured PBMCs. Cryopreserved PBMCs (1 x 10^6^ cells/ml) isolated from HTLV-1^+^ donors were cultured alone or co-cultured with allogeneic NK cells (4 x 10^6^ cells/ml) in the presence or absence of humanized anti-gp46 ADCC-inducing mAb (hu-LAT-27) for 2 days, and the number of CD4^+^Tax^+^ T cells was calculated by FCM. **(A)** FCM data on the case of an aggressive type of ATL patient (lymphoma type: ATL-1) showing a decrease in the number of CD4^+^Tax^+^ T cells by ADCC. **(B)** Decrease in the number of CD4^+^Tax^+^ T cells by ADCC in three of aggressive ATL and one AC. The number of CD16^+^CD56^+^ NK cells in the PBMCs from patients with lymphoma type ATL (Lymphoma type ATL-1), acute ATL (ATL-2 and ATL-3), and asymptomatic AC-4 were 200, 39,400, 500, and 92,000/well, respectively, as determined by FCM. Experiments were performed in duplicate. Statistical analysis was performed using Wilcoxon matched-pairs singed rank test.

Finally, we evaluated the role of anti-gp46 ADCC in primary T cells isolated from three aggressive ATL patients (one from lymphoma type ATL and two from acute type ATL) and one AC. PBMCs were co-cultured with allogeneic NK cells at an effector to target cell ratio of 4 in the presence or absence of a humanized anti-gp46 ADCC-inducing mAb (hu-LAT-27) for 2 days, and the number of CD4^+^Tax^+^ T cells was calculated as shown in **Figure 5A**. **Figure 5B** showed that a combination of hu-LAT-27 and NK cells significantly reduced the number of CD4^+^Tax^+^ cells in the PBMCs in comparison to NK cell alone. These results show that fresh ATL leukemic cells expressing Tax antigen were susceptible to ADCC.

## Discussion

In the present cross-sectional study using a large number of plasma samples collected in Okinawa, we showed that the levels of HTLV-1-neutralizing antibodies in the plasma were significantly lower in acute ATL patients than in ACs. In the past, similar observations were reported by Astier-Gin et al. using another neutralization method (a reporter gene inhibition assay to determine 50% inhibition) and samples from chronic and acute ATL patients (n=16) and ACs (n=41) among native inhabitants of Central Africa and the Caribbean area (22). Thus, it can be generalized that the decrease in the HTLV-1-neutralizing antibody titer is a possible accompanying biomarker of aggressive ATL.

Several questions remain to be addressed with the current data. Firstly, there was no significant difference in the neutralizing activity in chronic and smoldering ATL patients compared to ACs (**Figure 1**). However, a significant correlation existed between the anti-gp46 binding antibody level and the neutralization level in ACs, as well as in the acute and smoldering ATL but not in the chronic ATL (**Figure 2**). The reason for this is unclear; however, it is possible that the number of samples from chronic and smoldering ATL patients was small for a group analysis. Alternatively, as smoldering ATL and a part of chronic ATL are classified as indolent ATL types (23), the plasma titers of the neutralizing antibodies from patients with indolent type ATL may not be significantly different from those of ACs. Secondly, due to a limited number of samples, the correlation between ADCC–inducing activity and neutralizing antibody activity titer in each ATL subtype and ACs remains unclear. Thirdly, correlation studies between anti-gp46 antibody production and clinical laboratory data reflecting tumor volume remain to be undertaken. Our preliminary analysis indicated no correlation between anti-gp46 antibody titers and sIL-2R (sCD25) plasma levels (data not shown), which was in accordance with our recent findings that PVL and sIL-2R plasma levels correlate significantly (24). These data indicate that anti-gp46 antibody production is independent of ATL tumor volume. Further studies using more samples are in progress to address these questions.

Although the ADCC-inducing capability of purified IgG from acute ATL patients was comparable to that of ACs at a concentration of 100 µg/ml, based on the correlation between ADCC-inducing antibody levels and neutralizing antibody titers (**Figure 3B**), it is suggested that the total amount of ADCC-inducing antibodies might be lower in acute ATL patients than in ACs. Since ADCC mediated by a combination of anti-gp46 mAb and NK cells was able to reduce the number of Tax^+^ cells in freshly cultured PBMCs from ATL patients (**Figure 5**), it is suggested that the decreased levels of anti-HTLV-1 gp46 neutralizing and ADCC-inducing antibody concentrations might be related to the development of acute ATL. It seems likely that in the presence of a substantial number of CD16^+^NK cells in the blood or organs at an asymptomatic stage, HTLV-1-infected T cells that transiently become positive for HTLV-1 Tax antigen followed by gp46 expression will be eradicated instantly by the NK-mediated ADCC *via* the cell surface binding of anti-gp46 ADCC-inducing antibodies, as shown in **Figure 5**. Our preliminary study indicated that the addition of hu-LAT-27 (without NK cells) into PBMC cultures from the asymptomatic carrier (AC-4 in **Figure 5B**), wherein sufficient CD16^+^CD56^+^NK cells (10% of PBMCs) existed, did not induce ADCC (data not shown). The impaired function of the NK cells in the donor could be speculated from the cases reported for patients with ATL (25). Further studies are in progress to determine whether or not HTLV-1-specific ADCC-inducing ability is deficient in the NK cells of ATL patients and ACs.

The fact that such ADCC could not eradicate all Tax-expressing cells in primary cultured PBMCs from ATL patients (**Figure 5**) could suggest that Tax^+^ expressing cells are heterogeneous in the expression of cell surface gp46. It can be speculated that HTLV-1-specific CD8^+^ CTL directed against Tax and HBZ antigens (26) will cooperate together for the wide-range eradication of HTLV-1-expressing cells *in vivo*. However, we cannot exclude another possibility that immunodeficiency conditions in acute ATL patients (27–29) are the cause of the decreased antibody production. For a precise understanding, longitudinal follow-up studies with AC cohorts need to be performed.

An elevated HTLV-1 PVL is a hallmark of ATL and the sole risk factor for developing ATL in an asymptomatic state (30). However, it has been elusive whether anti-HTLV-1 antibody titers correlate with PVL. So far, using 45 samples from ACs, Akimoto et al. reported a positive correlation between PVL and antibody levels against purified HTLV-1 antigen plus synthetic Env peptides as determined by ECLIA (20). In accord, Burbelo et al. reported a correlation between PVL and antibody levels against recombinant HTLV-1 Env antigens as determined by LIPS assay in ACs but not in HAM/TSP patients (31). In addition, Matsumoto et al. showed a weak correlation between PVL and antibody levels against whole HTLV-1 antigens produced in HTLV-1-infected cell line as measured by CLIA (21). In contrast, no correlation was found between PVL and antibody levels against either recombinant Tax antigen as determined by ECLIA (20) or HBZ antigen as determined by LIPS assay (32) or ELISA (33). The present study is the first to show no significant correlation between PVL and anti-native gp46 antibody levels. Further studies with larger numbers of specimen will be required to solve the discrepancy on relationship between PVL and anti-HTLV-1 titers, in which the frequency of defective-type proviruses (34) in total PVL should be taken into account.

In conclusion, the present study indicated a possible role for anti-gp46 HTLV-1-neutralizing and ADCC-inducing antibodies in immuno-surveillance during the course of HTLV-1 infection. So far, it has been reported that the number and function of NK cells in aggressive ATL patients are low (25, 35), and that NK cells, but not monocytes, are the main effector cells in ADCC against both ATL leukemic cells in patients under the anti-CCR4 therapy (36) and HTLV-1-transformed cells in the presence of anti-gp46 mAb (14). Therefore, it might be worthwhile to investigate whether therapy that can elevate both HTLV-1-specific neutralizing and ADCC-inducing antibody levels and the number of functional NK cells would be beneficial for some patients with aggressive ATL by, for example, an adaptive immunization with a sufficient number of autologous CD16^+^NK cells along with the humanized anti-gp46 hu-LAT-27 mAb. Studies are in progress to explore an adequate method for *in vitro* expansion of CD16^+^NK cells from PBMCs of patients with acute ATL.

## Data availability statement

The original contributions presented in the study are included in the article/**Supplementary Material**. Further inquiries can be directed to the corresponding author.

## Ethics statement

This study was approved by the Human Institutional Review Board of the University of the Ryukyus (approval number:1470, 319-6 and 777-2). All blood samples and information were collected after obtaining written informed consent, according to the Declaration of Helsinki.The patients/participants provided their written informed consent to participate in this study.

## Author contributions

YuT and TF conceived the project and designed experiments. YuT performed experiments, data analysis and wrote the paper. TF established the ATL cohorts and supervised. RT performed ELISA, neutralization and ADCC experiments. NI performed PVL tests. YoT and MM managed the present cell and plasma banking. MH, TM, JU, KO, and HM collected clinical samples. All authors discussed the results and commented on the manuscript. All authors contributed to the article and approved the submitted version.

## Funding

This research was supported by AMED under grants 18fk0108060h0001 (YuT) and 20ek0109441h0001 (YuT), and grants from joint researches with Daiichi Sankyo Co., Ltd (YuT, and TF). The funder had no role in the design of the study, in the sample collection, analyses and interpretation of data, in the writing of the manuscript, or in the decision to publish the results.

## Acknowledgments

The authors would like to thank all of the blood donors who participated in this study, and to thank Editage (www.editage.com) for English language editing.

## Conflict of interest

The authors declare that the research was conducted in the absence of any commercial or financial relationships that could be construed as a potential conflict of interest.

## Publisher’s note

All claims expressed in this article are solely those of the authors and do not necessarily represent those of their affiliated organizations, or those of the publisher, the editors and the reviewers. Any product that may be evaluated in this article, or claim that may be made by its manufacturer, is not guaranteed or endorsed by the publisher.
