# Supplementary figures and images for: A protective role of HTLV-1 gp46-specific neutralizing and antibody-dependent cellular cytotoxicity-inducing antibodies in progression to adult T-cell leukemia (ATL)
Source: Front Immunol. 2022 Sep 13;13:921606. doi: 10.3389/fimmu.2022.921606 (PMC9513378; doi:10.3389/fimmu.2022.921606)

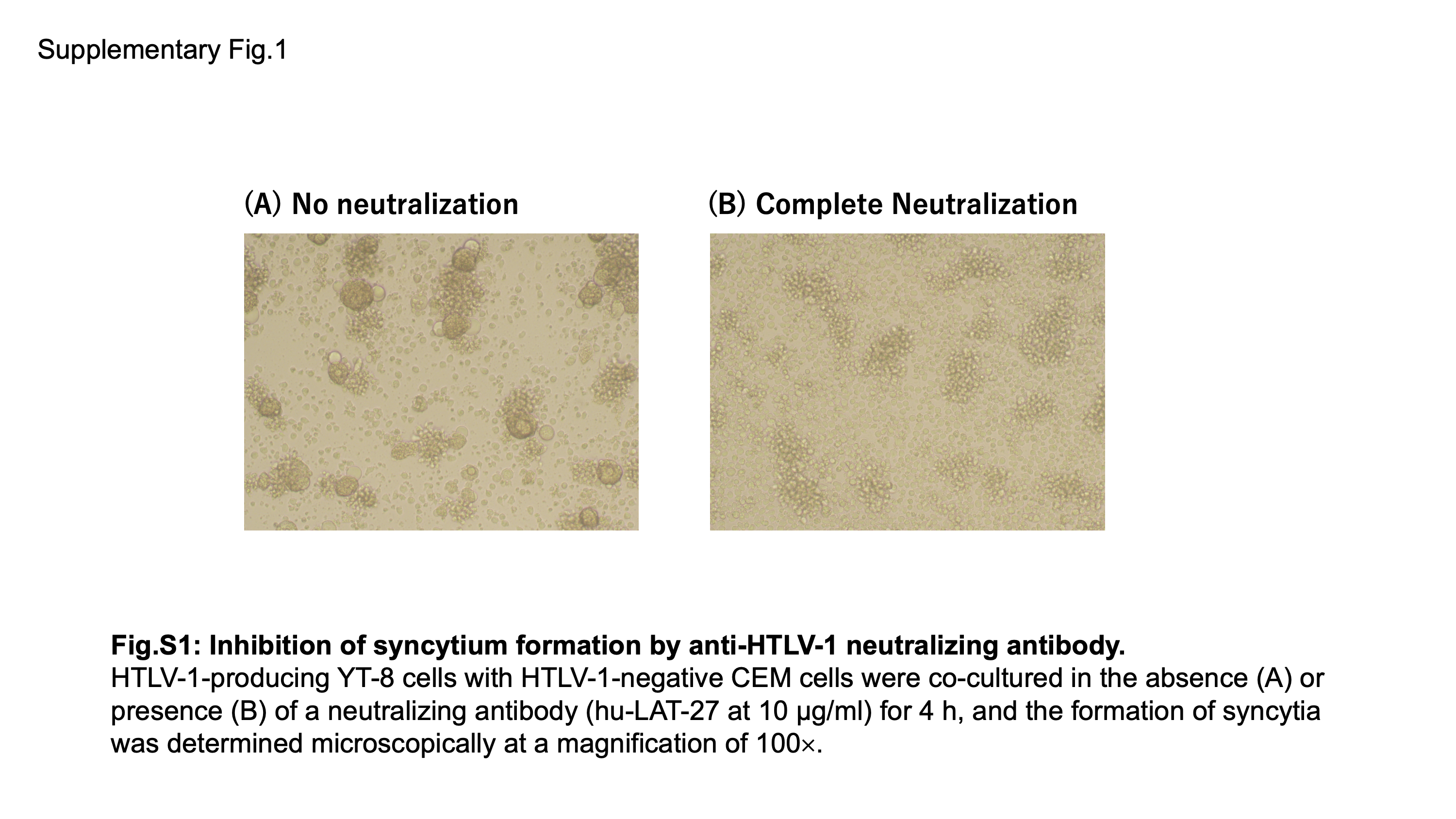

Supplement: Supplementary file 1 [file Image_1.tiff]

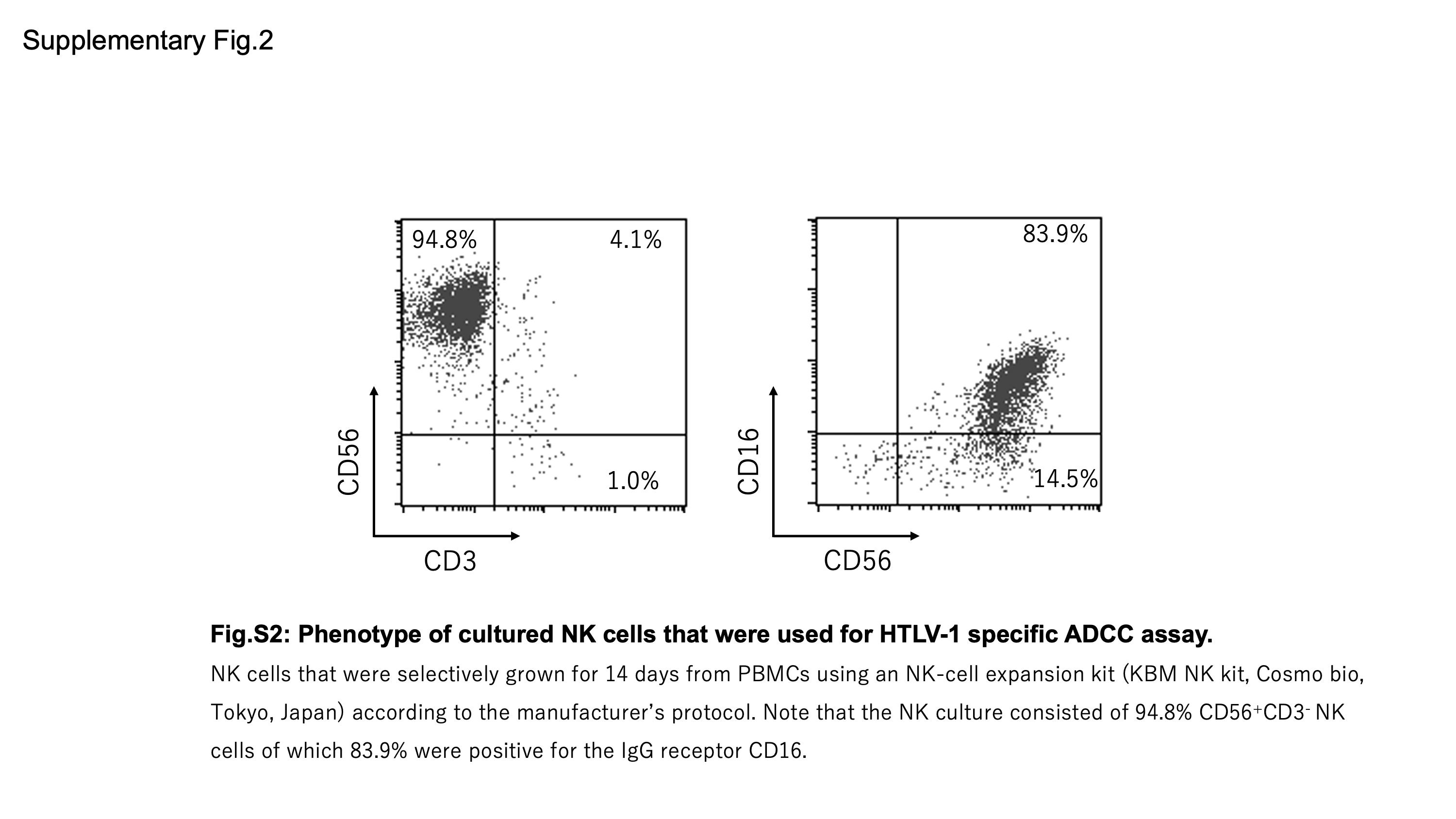

Supplement: Supplementary file 2 [file Image_2.tiff]

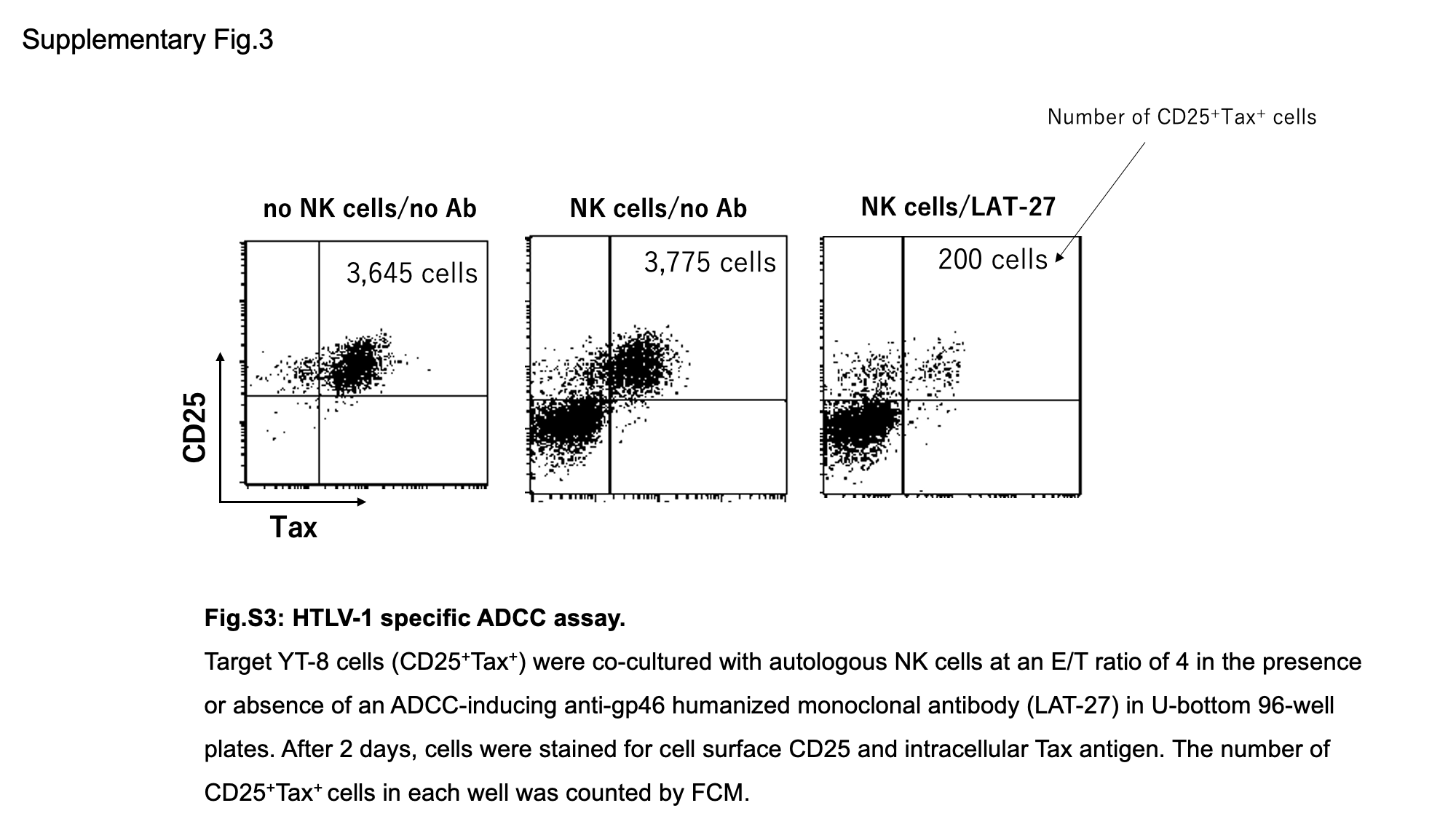

Supplement: Supplementary file 3 [file Image_3.tiff]

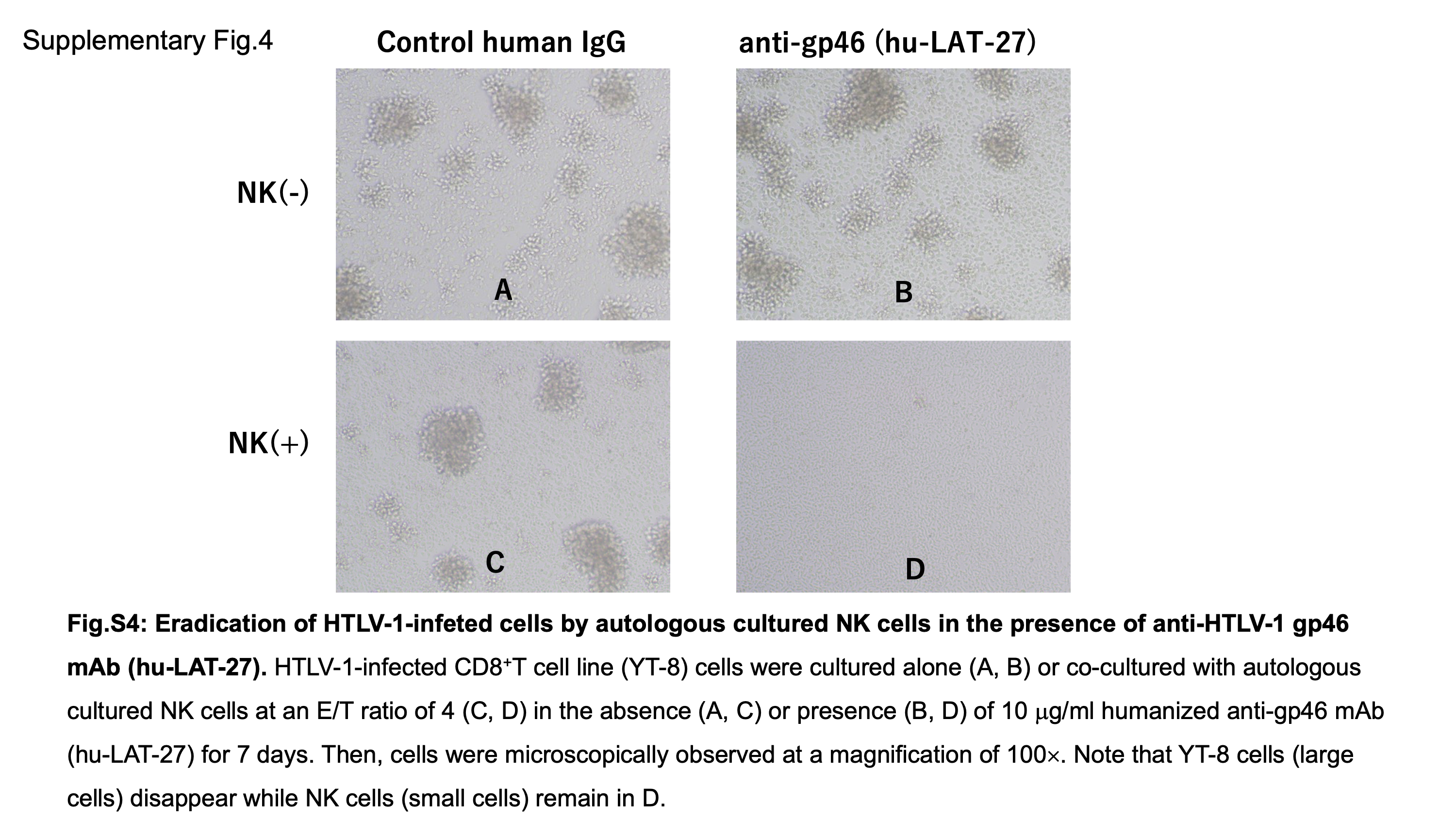

Supplement: Supplementary file 4 [file Image_4.tiff]
